# Association between high-risk metabolic dysfunction-associated steatohepatitis and ischemic heart disease: a nationally representative study

**DOI:** 10.1186/s12937-026-01342-6

**Published:** 2026-06-02

**Authors:** Bin Yuan, Xuehong Zheng, Zhimeng Wu, Lihan Zhu, Changchang Fang, Yi Xu, Jihao Xu, Huilei Zhao, Jingfeng Wang, Peng Yu, Xiao Liu, Zhiqiang Chen

**Affiliations:** 1 Department of Cardiovascular Surgery, the First Affiliated Hospital, Jiangxi Medical College, Nanchang University, Nanchang, Jiangxi Province China; 2 Department of Endocrinology and Metabolism, the Second Affiliated Hospital, Jiangxi Medical College, Nanchang University, Nanchang, Jiangxi Province China; 3 School of Rehabilitation, Jiangxi Medical College, Nanchang University, Nanchang, China; 4 Department of Physiology & Biomedical Sciences, Ischemic/Hypoxic Disease Institute, Seoul National University College of Medicine, Seoul, Korea; 5 Department of Medicine, Tseung Kwan O Hospital, New Territories, Hong Kong China; 6 Department of Anesthesiology, the People’s Hospital of Nanchang, Nanchang, China; 7 Institute for the Study of Endocrinology and Metabolism in Jiangxi Province, Nanchang, Jiangxi Province China; 8 Department of Cardiology, Sun Yat-sen Memorial Hospital of Sun Yat-sen University, Guangzhou, Guangdong China; 9 Guangdong Province Key Laboratory of Arrhythmia and Electrophysiology, Guangzhou, Guangdong China; 10 Department of Cardiology, the First Affiliated Hospital of Gannan Medical University, Ganzhou, China

**Keywords:** Ischemic heart disease, Metabolic dysfunction-associated steatohepatitis, National Health and Nutrition Examination Survey, FibroScan-AST (FAST) score, Transient elastography

## Abstract

**Background:**

The relationship between metabolic dysfunction-associated steatohepatitis (MASH) and ischemic heart disease (IHD) remains incompletely understood. This study investigated this association in the U.S. adults utilizing the FibroScan-AST (FAST) score, a validated non-invasive tool for identifying high-risk MASH.

**Methods:**

This cross-sectional study included 3,696 adults in the 2017–2018 National Health and Nutrition Examination Survey. We defined high-risk MASH as FAST score ≥ 0.35. We performed logistic regression to investigate the association between high-risk MASH and prevalent IHD. FAST was calculated by liver stiffness measurement (LSM), controlled attenuation parameter (CAP), and aspartate aminotransferase (AST).

**Results:**

The prevalence of IHD was significantly higher in the high-risk MASH group than in the non-high-risk group (10.5% vs. 5.8%). Both LSM (adjusted odds ratio (aOR) 1.84, 95% CI 1.28–2.65) and CAP (aOR 3.06, 95% CI 1.32–7.12) were independently associated with IHD after adjustment, whereas AST did not demonstrate a significant association (aOR 0.83, 95% CI 0.48–1.43). High-risk MASH was independently associated with IHD (aOR: 2.08; 95% CI: 1.18–3.65). Subgroup analysis suggested a potential sex-specific interaction (P for interaction = 0.035), with a pronounced association in males (aOR: 2.51; 95% CI: 1.32–4.75) but was not statistically significant in females (aOR: 0.36, 95% CI 0.07–1.27).

**Conclusion:**

High-risk MASH defined by the FAST score is independently associated with prevalent IHD in the U.S. population. Future prospective cohort studies are warranted to validate whether intervation of high-risk MASH improves cardiovascular outcomes.

**Supplementary Information:**

The online version contains supplementary material available at 10.1186/s12937-026-01342-6.

## Introduction

Ischemic heart disease (IHD) remains a leading cause of mortality worldwide, accounting for more than 9 million deaths annually [1, 2]. In the United States, approximately 720,000 new coronary events occur each year, with an estimated 18.2 million individuals affected by IHD, according to the American Heart Association [2]. Given the substantial burden, identifying novel clinical factors associated with IHD is crucial for comprehensive risk assessment and integrated management of the condition.

Metabolic dysfunction-associated steatotic liver disease (MASLD), formerly known as nonalcoholic fatty liver disease (NAFLD), has emerged as a global epidemic, affecting over 25% of the adult population [4, 6, 24]. Approximately 20% of individuals with MASLD progress to metabolic dysfunction-associated steatohepatitis (MASH), previously termed nonalcoholic steatohepatitis (NASH), which represents the active, progressive form of MASLD characterized by hepatocyte ballooning and inflammation [5]. MASH is not only strongly associated with adverse hepatic outcomes, such as cirrhosis and hepatocellular carcinoma [7], but is also increasingly recognized as a systemic metabolic driver.

A well-established link exists between MASLD and cardiovascular disease (CVD), with CVD being recognized as the primary cause of death in individuals with MASLD [3, 8, 9]. Indeed, this complex interplay is increasingly viewed as an emerging paradigm, where the burden of metabolic dysfunction and steatotic liver disease casts a long shadow over cardiovascular health [10]. While numerous studies have linked broad MASLD to IHD independent of traditional metabolic risk factors [11, 12], the specific relationship between high-risk MASH and IHD remains understudied. High-risk MASH patients, characterized by concurrent active steatohepatitis and significant fibrosis, theoretically bear a higher inflammatory and metabolic burden than those with simple steatosis. Consequently, they may be particularly susceptible to accelerated atherosclerosis and adverse cardiovascular outcomes.

However, historical reliance on invasive liver biopsy as gold standard has limited large-scale epidemiological investigation into high-risk MASH. Consequently, there is a critical need to utilize validated, non-invasive diagnostic methods capable of identifying this high-risk subgroup. The FibroScan-AST (FAST) score has emerged as a validated non-invasive algorithm specifically developed to identify patients with high-risk MASH (formerly high-risk NASH), defined by elevated NAFLD activity score (NAS ≥ 4) and significant fibrosis (stage F ≥ 2) [6, 13]. Leveraging this tool, the present study utilized a nationally representative cohort to estimate the odds of IHD among individuals with FAST score-defined high-risk MASH and to assess the association between high-risk MASH and prevalent IHD.

## Methods

### Participants

The National Health and Nutrition Examination Survey (NHANES) is a biennial program designed to assess the health and nutritional status of the U.S. population. By integrating structured interviews, comprehensive physical examinations, and laboratory assessments, the NHANES provides nationally representative health statistics. This cross-sectional study utilized data from the 2017–2018 NHANES cycle, which included 9245 participants selected through a complex, multistage stratified sampling design. Detailed information regarding the NHANES methodology and data collection procedures is available on its official website. This study is presented in accordance with the STROBE reporting checklist.

### Measurement

Anthropometric measurements were conducted following standardized NHANES protocols. Vibration-controlled transient elastography (VCTE) was performed via an ultrasound device (FibroScan model 502 V2 Touch) to assess the controlled attenuation parameter (CAP) and liver stiffness measurement (LSM). Blood pressure (BP) was recorded as the mean value of three consecutive seated measurements obtained after the participants had rested quietly for five minutes. Weight and height were obtained when people attended a physical examination at a mobile health center. Body mass index (BMI) was calculated as weight (kg) divided by height squared (m²). Blood biochemistry parameters, including C-reactive protein (CRP), total serum protein, creatinine, aspartate aminotransferase, alanine aminotransferase, and triglyceride (TG) levels, were measured via a Roche Cobas 6000 analyzer (c501 module) [14, 15]. The estimated glomerular filtration rate (eGFR) was calculated via the Chronic Kidney Disease Epidemiology Collaboration (CKD-EPI) equation [16]. Glycated hemoglobin (HbA1c) was measured using a Tosoh automated glycohemoglobin analyzer based on high-performance liquid chromatography. Total cholesterol (TC) and high-density lipoprotein cholesterol (HDL-C) were quantified via enzymatic methods in participants who fasted for 8 to less than 24 h. All measurements were conducted at the same examination visit at NHANES Mobile Examination Centers (MECs) by trained health technicians following strict quality control procedures. The detailed sample collection and testing methodologies are available in the NHANES Laboratory Procedures Manual [17].

### FAST score and high-risk MASH definitions

High-risk MASH, clinically defined as active fibrotic steatohepatitis, is characterized by a NAFLD activity score (NAS) ≥ 4 and fibrosis stage (F) ≥ 2. To identify this specific phenotype non-invasively, we utilized the FAST score, a validated algorithm derived from LSM, CAP, and aspartate aminotransferase (AST) levels [13]. The FAST score was calculated using the following equation:

$$\:\mathrm{FAST}=\frac{\mathrm{e}^{-1\cdot65+1\cdot07\times\:ln\left(\mathrm{LSM}\right)+2\cdot66\ast10^{-8}\times\:\mathrm{CAP}^3-63\cdot3\times\:\mathrm{AST}^{-1}}}{1+\mathrm{e}^{-1\cdot65+1\cdot07\times\:ln\left(\mathrm{LSM}\right)+2\cdot66\ast10^{-8}\times\:\mathrm{CAP}^3-63\cdot3\times\:\mathrm{AST}^{-1}}}$$

Based on the original derivation and validation study, a FAST score cutoff of 0.35 provides a sensitivity of 0.90 or greater and a specificity of 0.67 or greater, yielding a positive predictive value (PPV) of 0.83 and a negative predictive value (NPV) of 0.85 [13]. Consequently, our primary definition for high-risk MASH utilized a FAST score cutoff of ≥ 0.35. This threshold was selected to prioritize sensitivity (sensitivity ≥ 0.90), thereby capturing the broadest spectrum of high-risk MASH individuals suitable for population-based screening. Complementarily, we applied the stricter cutoff of ≥ 0.67 (specificity ≥ 0.90) to validate the association within a cohort characterized by higher diagnostic certainty. Furthermore, to further evaluate the robustness of the association between high-risk MASH and IHD, we performed a sensitivity analysis using a lower FAST score cutoff of 0.25. This FAST score has been previously validated and used in many reports [18–20].

The CAP and LSM have been previously used to define steatotic liver disease and clinically significant fibrosis (CSF; fibrosis stage ≥ 2) in a U.S.-based cohort [21]. In this study, consistent with the MASLD criteria, significant steatosis was defined as a CAP value ≥ 285 dB/m, and CSF was defined as an LSM ≥ 8.6 kPa. Circulating AST levels were incorporated into the model as a continuous marker of active hepatocellular injury, reflecting the inflammatory component of steatohepatitis.

### Definition of comorbidities and IHD

Individuals were considered to have a history of IHD if they had ever been diagnosed by a physician with angina pectoris, coronary heart disease, myocardial infarction, or heart attack. The validity of self-reported cardiovascular outcomes in large-scale epidemiological surveillance has been well established, demonstrating high specificity and substantial concordance with medical records [22]. This definition represents a standard methodology widely adopted in high-impact analyses of NHANES data [23].

Hypertension was defined as a systolic blood pressure (SBP) ≥ 140 mmHg, a diastolic blood pressure (DBP) ≥ 90 mmHg, or self-reported hypertension. Diabetes was identified on the basis of a self-reported diagnosis by a healthcare professional, a fasting plasma glucose level ≥ 126 mg/dL, or a glycated hemoglobin (HbA1c) level ≥ 6.5%. Smoking status was categorized into three groups: (1) never, defined as individuals who had smoked fewer than 100 cigarettes in their lifetime; (2) former, who had smoked more than 100 cigarettes in their lifetime but were not current smokers; and (3) current, who had smoked more than 100 cigarettes in their lifetime and continued to smoke at the time of the survey. Based on the Physical Activity Guidelines for Americans, the physical activity categories were established according to the recommended amount of moderate-intensity to vigorous-intensity activity that should be performed weekly: (1) below guidelines, which indicates less than 150 min per week; (2) meets guidelines, which means 150 to 300 min per week; and (3) exceeds guidelines, which means more than 300 min per week. Demographic data, including age, race/ethnicity, and sex, were obtained through interviews. Obesity was defined as a body mass index (BMI) ≥ 30 kg/m².

In this study, heart failure (HF) status was ascertained based on self-reported medical diagnoses. Participants were classified as having prevalent HF if they answered affirmatively to the question: “Have you ever been told by a doctor or other health professional that you had congestive heart failure?” Correspondingly, stroke history was identified using a parallel standardized question: “Have you ever been told by a doctor or other health professional that you had a stroke?”

### Statistical analysis

This analysis was conducted in accordance with NHANES analytic guidelines to account for the complex, multistage survey design and to generate nationally representative estimates. Sampling weights, strata, and primary sampling units were incorporated in all analyses, including the primary logistic regressions, subgroup analyses, and sensitivity analyses. Weighted means with 95% confidence intervals are reported for continuous variables, whereas weighted proportions with 95% CIs are provided for categorical variables. Differences in participant characteristics based on a FAST score ≥ 0.35 were assessed via t tests for continuous variables and chi-square tests for categorical variables. Additionally, we estimated the weighted prevalence of IHD among individuals with high-risk MASH and compared the IHD incidence across different age groups (< 55 years, 55–69 years, and 70–85 years) within this population. Multivariate logistic regression analysis was performed to estimate the associations between high-risk MASH and IHD, as well as other comorbidities, by calculating odds ratios (ORs) with 95% confidence intervals. Histograms were used to display the distributions of LSM, AST, CAP, and the FAST score. To assess the independent associations between individual FAST score components and IHD, we performed survey-weighted logistic regression analyses. LSM, AST, and CAP were log-transformed to address skewness.

Three models were constructed: Model 1, adjusted for sex, race and age; Model 2, further adjusted for smoking status and physical activity; and Model 3, adjusted for BMI, diabetes mellitus, hypertension, antihypertensive drugs, glucose-lowering drugs, cholesterol-lowering drugs, eGFR, TC, HDL-C, ALT, total serum protein, and CRP. To minimize residual confounding and maximize information retention, covariates were rigorously defined based on biological plausibility. Continuous variables, including age, BMI, eGFR, TC, HDL-C, ALT, total serum protein, and CRP, were entered directly into the models. Categorical variables were sex, race, smoking status, diabetes mellitus status, hypertension status, physical activity use of antihypertensive drugs, use of glucose-lowering drugs, and use of cholesterol-lowering drugs.

Missing data were present in a small proportion of covariates, including BMI (0.81%), antihypertensive drugs (0.22%), glucose-lowering drugs (0.11%), cholesterol-lowering drugs (0.62%), TC (0.11%), HDL-C (0.11%), and CRP (0.19%). To minimize bias and preserve the national representativeness of the sample, we performed multiple imputation using the Markov chain Monte Carlo (MCMC) method. The imputation model included the exposure, outcome, and all adjustment covariates to preserve correlation structures. Five imputed datasets were generated, and logistic regression estimates were pooled using Rubin’s rules [25].

To further strengthen the robustness of our findings, we performed a sensitivity analysis using alternative cutoff values for the FAST score of 0.25 to assess the consistency of the association between high-risk MASH and IHD.

To estimate potential effect modification, subgroup analyses were performed on the basis of baseline variables, including sex, age (< 65 vs. ≥ 65 years), body mass index (BMI < 30 vs. ≥ 30 kg/m²), presence or absence of diabetes, presence or absence of hypertension, estimated glomerular filtration rate (eGFR < 60 vs. ≥ 60 mL/min/1.73 m²), total cholesterol (TC < 200 vs. ≥ 200 mg/dL), and smoking status (never, former, or current). Interaction effects were assessed via log-likelihood ratio tests to compare differences in effect estimates. A two-tailed p value of < 0.05 was considered statistically significant. All statistical analyses were performed via R (version 4.1.2) and SPSS (version 28.0).

## Results

### Characteristics of participants

**Fig. 1:**
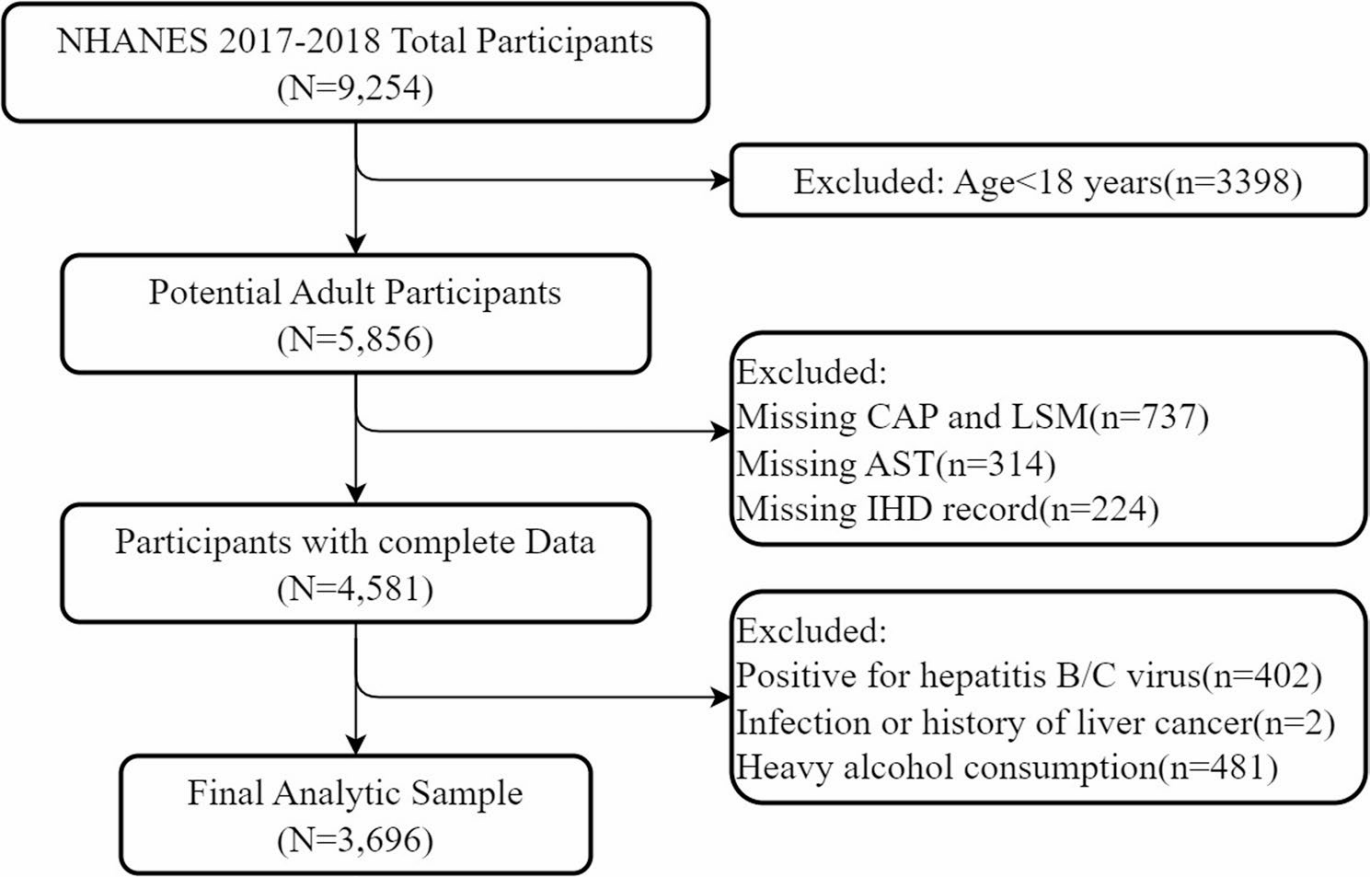
Participant selection flowchart. This flowchart illustrates the participant selection process from the NHANES 2017–2018 cohort. The diagram shows the sequential exclusion of participants based on age criteria and three specific exclusion criteria: insufficient data for FAST score calculation or missing IHD information, presence of viral hepatitis or liver cancer, and heavy alcohol consumption. The final analytical cohort consisted of 3,696 U.S. adults included in the cross-sectional analysis of the association between high-risk MASH and IHD. *Abbreviations*: NHANES, National Health and Nutrition Examination Survey; CAP, controlled attenuation parameter; LSM, liver stiffness measure; AST, aspartate aminotransferase; IHD, ischemic heart disease; HBV, hepatitis B virus; HCV, hepatitis C virus; MASH, metabolic dysfunction-associated steatohepatitis

As shown in Fig. 1, among the 9,254 participants in the NHANES 2017–2018 cycle, 5,856 aged ≥ 18 years were included. On the basis of the following criteria, 2,160 participants were excluded from the study: (1) insufficient data to calculate the FAST score or missing questionnaire-based data on IHD; (2) positive serum markers for hepatitis B or C virus infection or a history of liver cancer; and (3) heavy alcohol consumption (≥ 20 g/day for women and ≥ 30 g/day for men). Consequently, 3,696 participants were included in the final analysis.

A sensitivity analysis revealed significant differences in key characteristics between the included (*n* = 3,696, 69.28%) and excluded (*n* = 2,160, 30.72%) participants (Table S1). Compared with the excluded participants, those included in the analysis were older; had a higher BMI and HbA1c; and had lower HDL-C, eGFR, and aspartate amino transferase levels. Additionally, the included participants were more likely to have diabetes and obesity.

The baseline characteristics of the study participants are presented in Table 1. The cohort comprised non-Hispanic White (62.49%), non-Hispanic Black (10.53%), Mexican American (9.68%), other Hispanic (7.23%), and individuals from other racial/ethnic backgrounds (10.08%). Males accounted for 47.41% of the cohort, 38.46% had hypertension, and 15.50% had diabetes. The mean age of the participants was 48.27 years. Among the total study population, 57.34% had MASLD, and 8.19% had an LSM ≥ 8.6 kPa, indicative of CSF. Participants with high-risk MASH were more likely to be male; have hypertension, diabetes, obesity, and a history of smoking (former smokers) and be more likely to use antihypertensive and glucose-lowering medications more frequently. In terms of the measured variables, individuals with high-risk MASH presented higher BMIs; SBP, DBP, HbA1c, TG, ALT, AST, total serum protein, CRP, CAP, and LSM; and lower HDL-C levels. No significant differences were observed across the MASH groups in terms of age, race/ethnicity, physical activity, TC, eGFR, or the use of cholesterol-lowering medications.

**Table 1:** Baseline characteristics of individuals grouped by MASH status in adult Americans from the National Health and Nutrition Examination Survey 2017–2018

Variables	Whole cohort, *n* = 3696	FAST score < 0.35, *n* = 3432	FAST score ≥ 0.35, *n* = 264	*p*
Demographic variables				
Age, year	48.2(46.8,49.6)	48.3(46.8,49.7)	47.9(45.3,50.4)	0.746
Male, sex	47.4(45.0,49.8)	46.1(45.5,48.7)	65.8(56.3,74.2)	0.002
Race/ethnicity				0.177
Mexican American	9.68(6.55,14.08)	9.43(6.33,13.84)	13.25(8.38,20.31)	
Other Hispanic	7.23(5.56,9.33)	7.12(5.45,9.26)	8.65(5.46,13.44)	
Non-Hispanic White	62.49(57.03,67.65)	62.72(57.19,67.94)	59.06(47.57,69.64)	
Non-Hispanic Black	10.53(7.55,14.50)	10.75(7.82,14.61)	7.34(3.48,14.85)	
Others	10.08(7.57,13.32)	9.97(7.49,13.16)	11.69(7.20,18.43)	
Comorbidities				
Physical activity				0.570
Below guidelines	33.54(31.33 ,35.82)	33.45(31.20 ,35.78)	34.81(26.23 ,44.51)	
Meets guidelines	14.51(12.98 ,16.20)	14.74(13.13 ,16.51)	11.31(6.46 ,19.07)	
Exceeds guidelines	51.95(49.33 ,54.56)	51.81(49.23 ,54.38)	53.88(44.70 ,62.80)	
Smoking status				0.002
Current	15.62(13.01,18.63)	15.76(13.00,18.99)	13.53(9.15,19.56)	
Former	24.44(21.69,27.42)	23.61(20.98,26.45)	36.53(28.68,45.16)	
Never	59.94(56.38,63.39)	60.63(57.14,64.02)	49.94(41.92,57.97)	
Hypertension	38.46(34.36,42.73)	37.22(33.24,41.38)	56.40(46.87,65.48)	< 0.001
Systolic BP, mm Hg	121.46(120.27,122.65)	121.10(119.94,122.26)	126.59(123.68,129.49)	0.001
Diastolic BP, mm Hg	73.75(72.86,74.63)	73.28(72.42,74.14)	80.31(79.05,81.58)	< 0.001
BMI, kg/m2	30.03(29.46,30.60)	29.51(28.97,30.05)	37.51(35.90,39.12)	< 0.001
Obesity	44.18(40.05,48.39)	41.54(37.76,45.42)	82.36(74.05,88.42)	< 0.001
Diabetes	15.50(14.03,17.09)	13.89(12.50,15.40)	38.71(29.98,48.23)	< 0.001
Medication use				
Antihypertensive drugs	23.67 (20.64 ,26.99)	23.01 (19.92 ,26.42)	33.13 (27.49 ,39.30)	< 0.001
Glucose-lowering drugs	11.09 (10.26 ,11.98)	10.02 (9.14 ,10.97)	26.56 (20.38 ,33.80)	< 0.001
Cholesterol-lowering drugs	18.28 (15.31 ,21.68)	18.35 (15.39 ,21.75)	17.17 (10.42 ,26.98)	0.752
Laboratory variables				
HbA1c (%)	5.72(5.68,5.76)	5.68(5.64,5.72)	6.32(6.12,6.53)	< 0.001
Total cholesterol, mg/dL	188.41(184.78,192.04)	188.25(184.53,191.98)	190.67(182.50,198.84)	0.575
Triglyceride	144.85(137.99,151.71)	139.39(132.89 ,145.89)	222.95(180.30 ,265.60)	0.002
HDL-C, mg/dL	52.52(51.72,53.33)	53.18(52.41,53.94)	43.11(41.18,45.05)	< 0.001
eGFR	95.96(94.21,97.70)	95.78(93.96,97.60)	98.50(96.09,100.91)	0.066
AST, IU/L	21.34(20.92,21.77)	19.99(19.58,20.40)	40.87(37.48,44.25)	< 0.001
ALT, IU/L	22.42(21.69,23.15)	20.35(19.76,20.94)	52.37(48.02,56.71)	< 0.001
Serum total protein (g/dL)	7.09(7.06,7.13)	7.08(7.05,7.12)	7.23(7.16,7.30)	0.001
CRP	3.95(3.59,4.31)	3.83(3.45,4.21)	5.59(5.02,6.15)	< 0.001
Fibroscan				
CAP (dB/m)	264.49(260.85,268.12)	258.78(255.38,262.18)	346.91(338.63,355.19)	< 0.001
MASLD	57.34 (54.25, 60.43)	54.7 (51.53, 57.95)	94.95 (92.45, 97.46)	< 0.001
LSM (kPa)	5.83(5.62,6.03)	5.24(5.10,5.39)	14.24(12.66,15.82)	< 0.001
CSF	8.19(6.58,10.16)	4.83(3.70,6.29)	56.72(48.71,64.40)	< 0.001

Data are expressed as weighted means (95% confidence intervals) for continuous variables and proportions for categorical variables. P values are derived from weighted tests (continuous) or chi-square tests (categorical)

*Abbreviations*: ,MASH, metabolic dysfunction-associated steatohepatitis; FAST, FibroScan-AST; BMI, body mass index; BP, blood pressure; HbA1c, glycated hemoglobin; HDL-C, high-density lipoprotein cholesterol; eGFR, estimated glomerular filtration rate; AST, aspartate aminotransferase; ALT, alanine aminotransferase; CRP, C-reactive protein; CAP, controlled attenuation parameter; LSM, liver stiffness measure; MASLD, metabolic dysfunction-associated steatotic liver disease; CSF, clinically significant fibrosis

### Distributional characteristics and independent associations of FAST score components

**Fig. 2:**
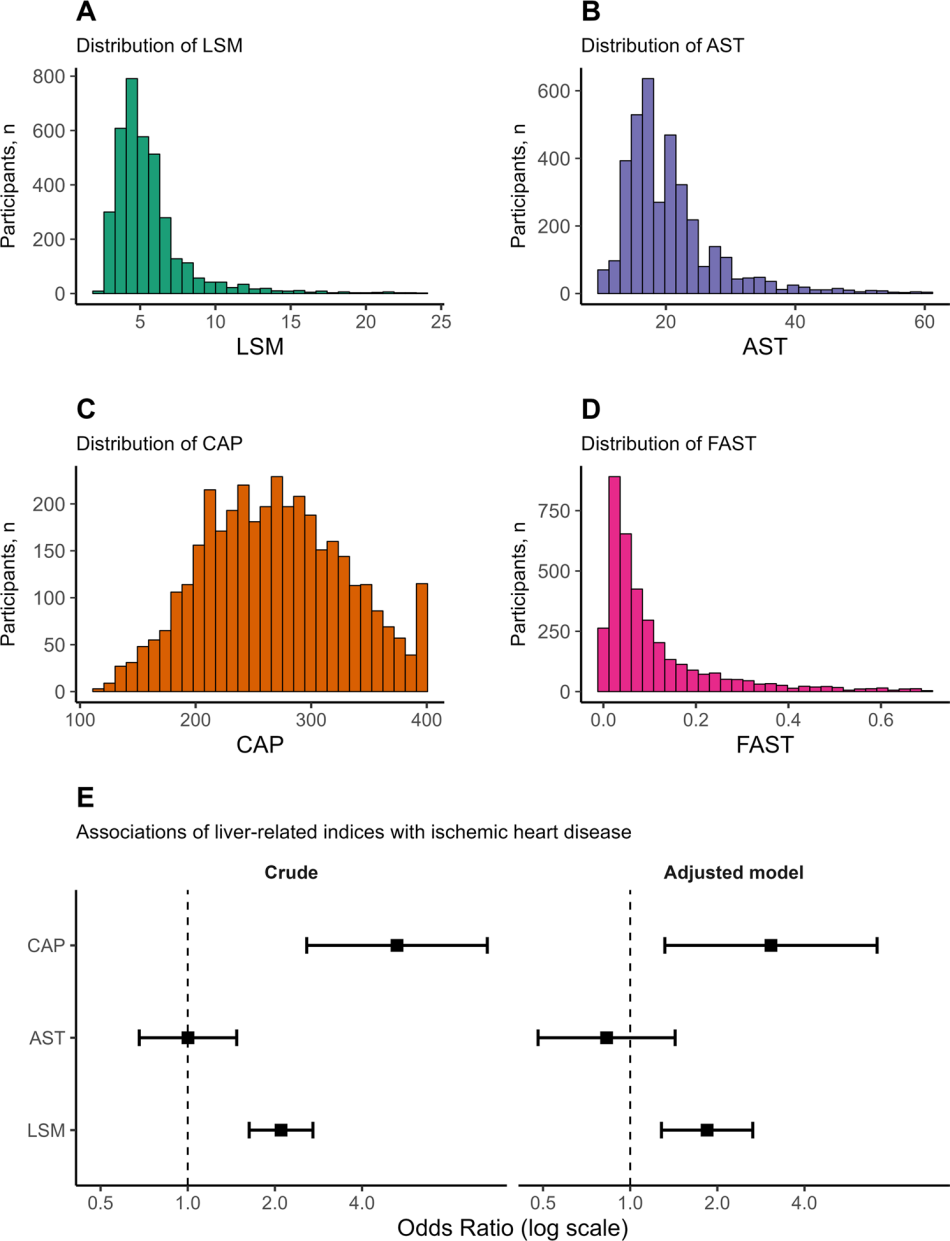
Distributions of liver-related indices and their associations with IHD. **A**–**D** Histograms showed the distributions of LSM, AST, CAP, and FAST score after exclusion of values below the 1st percentile and above the 99th percentile. **E** Forest plot displays ORs and 95% CIs for the associations between LSM, AST, and CAP with IHD under crude and adjusted models. The adjusted model was adjusted for age, sex, and race, accounting for the complex survey design. *Abbreviations*: IHD, ischemic heart disease; LSM, liver stiffness measure; AST, aspartate aminotransferase; CAP, controlled attenuation parameter; ORs, odds ratios; CIs, confidence intervals

Fig. 2A-D depicts the distributional characteristics of the FAST score and its individual components. The median (IQR) values were 5.0 (4.0–6.2) kPa for LSM, 19 (16–23) U/L for AST, 267 (220–312) dB/m for CAP, and 0.060 (0.028–0.133) for the FAST score.

Fig. 2E presents the associations between these individual components and prevalent IHD. LSM was significantly associated with higher odds of IHD (adjusted odds ratio (aOR) 1.84, 95% CI 1.28–2.65), persisting even after controlling for age, sex, and race. A similar independent association was observed for CAP (aOR 3.06, 95% CI 1.32–7.12), although the effect size was attenuated compared to the crude analysis (OR 5.28, 95% CI 2.58–10.83). AST did not demonstrate a significant independent association with IHD in either the crude (OR 1.00, 95% CI 0.68–1.48) or adjusted models (aOR 0.83, 95% CI 0.48–1.43).

### Associations between high-risk MASH and IHD

**Fig. 3:**
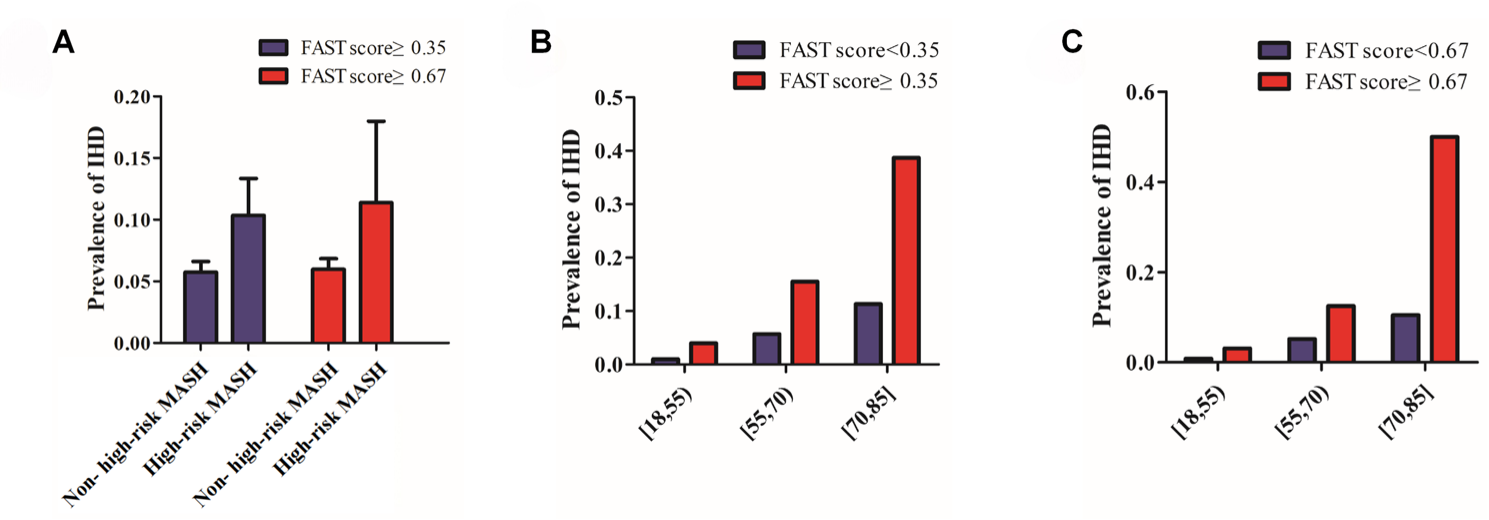
Prevalence of IHD stratified by FAST score categories. **A** Comparison of IHD prevalence between high-risk MASH and non-high-risk groups using both the primary high-sensitivity cutoff (FAST score ≥ 0.35) and the confirmatory high-specificity cutoff (FAST score ≥ 0.67). **B** Prevalence of IHD stratified by age within the primary high-risk MASH group (FAST score ≥ 0.35). **C** Prevalence of IHD stratified by age within the strict high-specificity group (FAST score ≥ 0.67). *Abbreviations*: MASH, metabolic dysfunction-associated steatohepatitis; FAST, FibroScan-AST; IHD, ischemic heart disease

Using our primary high-sensitivity definition (FAST score ≥ 0.35), the prevalence of IHD was observed to be higher in the high-risk MASH individuals (10.5%, 95% CI: 5.6–18.9%) than in the non-high-risk individuals (5.8%, 95% CI: 4.2–8.0%). A similar trend was observed when applying the high-specificity cutoff (FAST score ≥ 0.67), with a higher prevalence observed in individuals with high-risk MASH (11.5%, 95% CI: 3.0–35.9%) than in those with non-high-risk MASH (6.0%, 95% CI: 4.4–8.2%) (Fig. 3A). Furthermore, across all age subgroups, participants with high-risk MASH presented a higher prevalence of IHD (Fig. 3B and C).

There was increased odds of IHD and high-risk MASH in the unadjusted analysis (crude model, OR: 2.10; 95% CI: 1.36–3.17). The results remained significant in all adjusted models (model 1: aOR: 1.91; 95% CI: 1.23–2.89; model 2, aOR: 1.85, 95% CI: 1.18–2.82; Model 3 aOR: 2.08; 95% CI: 1.18–3.65) (Table 2). Furthermore, a sensitivity analysis utilizing multiple imputation to address missing data demonstrated a consistent and statistically significant association between high-risk MASH and IHD (pooled OR: 1.86; 95% CI: 1.08–3.20) (Table S2).

**Table 2:** Association of high-risk MASH (FAST score ≥ 0.35) with ischemic heart disease in adult Americans from the National Health and Nutrition Examination Survey 2017–2018

	Cases/*N*	Odds ratio	95% confidence interval	*P* value
FAST score < 0.35	244/3432	Ref	Ref	Ref
FAST score ≥ 0.35	31/264			
Crude		2.10	1.36,3.17	0.001
Model 1		1.91	1.23,2.89	0.003
Model 2		1.85	1.18,2.82	0.005
Model 3		2.08	1.18,3.65	0.011

Model 1 was adjusted for sex, race, and age; Model 2 was adjusted for sex, race, age, physical activity and smoking status; Model 3 was adjusted for sex, race, age, physical activity, smoking status, BMI, diabetes mellitus, hypertension, antihypertensive drugs, glucose-lowering drugs, cholesterol-lowering drugs, eGFR, TC, HDL-C, ALT, total serum protein, and CRP

*Abbreviations:* MASH, metabolic dysfunction-associated steatohepatitis; FAST, FibroScan-AST; BMI, body mass index; eGFR, estimated glomerular filtration rate; TC, total cholesterol; HDL-C, high-density lipoprotein cholesterol; ALT, alanine aminotransferase; CRP, C-reactive protein

In the sensitivity analysis using an alternative cutoff value of 0.25 for the FAST score (Table S3), there were greater odds of IHD and high-risk MASH (model 1: aOR: 1.45; 95% CI: 1.01–2.07; model 2, aOR: 1.77, 95% CI: 1.15–2.73; Model 3: aOR: 1.65; 95% CI: 1.03–2.64).

### Subgroup analyses and sensitivity analyses

**Fig. 4:**
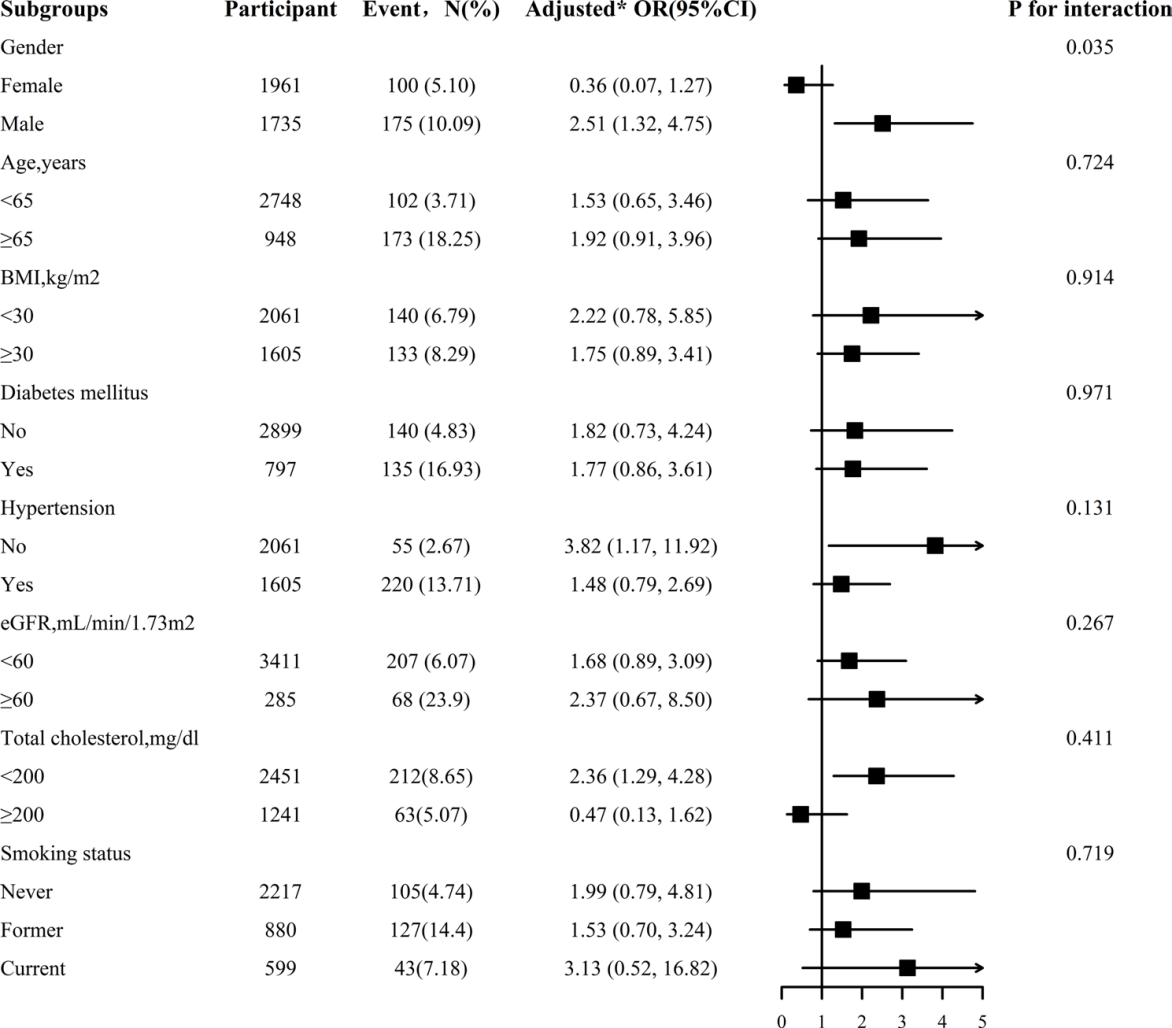
Association between high-risk MASH (FAST score ≥ 0.35) and IHD in various subgroups*. *Adjusted for sex, race, age, physical activity, smoking status, BMI, diabetes mellitus, hypertension, antihypertensive drugs, glucose-lowering drugs, cholesterol-lowering drugs, eGFR, TC, HDL-C, ALT, total serum protein, and CRP. (Variables were not adjusted if they were regarded as a stratified factor in subgroups). *Abbreviations*: BMI, body mass index; eGFR, estimated glomerular filtration rate; MASH, metabolic dysfunction-associated steatohepatitis; FAST, FibroScan-AST; IHD, ischemic heart disease; HDL-C, high-density lipoprotein cholesterol; ALT, alanine aminotransferase; CRP, C-reactive protein

Stratified analyses were performed to explore the relationship between high-risk MASH (FAST score ≥ 0.35) and IHD (Fig. 4). Our findings suggest a potential sex interaction in this association (P for interaction = 0.035). Specifically, the association was pronounced in males (aOR: 2.51; 95% CI: 1.32–4.75) but was not statistically significant in females (aOR: 0.36; 95% CI: 0.07–1.27). There were no significant interactions in other subgroups, including age (< 65 vs. ≥ 65 years), BMI (< 30 vs. ≥ 30 kg/m^2^), diabetes (yes vs. no), hypertension (yes vs. no), eGFR (< 60 vs. ≥ 60 mL/min/1.73 m^2^), and smoking status (never vs. former vs. current) (P interaction > 0.1).

### Associations between high-risk MASH and HF and stroke

We further explored the associations between high-risk MASH and two major cardiovascular diseases: HF and stroke. As shown in Table S4, high-risk MASH was significantly associated with a likelihood of HF (aOR: 3.05; 95% CI: 1.44–6.24). However, no significant association was observed between high-risk MASH and stroke (aOR: 0.80; 95% CI: 0.33–1.73) after adjusting for potential confounders (Model 3).

## Discussions

### Major findings

High-risk MASH is recognized as a major risk factor for disease progression to cirrhosis, hepatocellular carcinoma, liver transplantation, and liver-related mortality [13, 26]. While the association between a broad spectrum of MASLD (formerly NAFLD) and cardiovascular risk is well documented [27–29], the specific relationship between high-risk MASH, characterized by progressive fibrosis and active inflammation, and IHD in the general population remains less understood. Specifically, it is unclear whether high-risk MASH is associated with an elevated risk of IHD events.

A Swedish cohort study of 129 patients with elevated liver enzymes and biopsy-confirmed NAFLD (the diagnostic predecessor of MASLD) reported that NASH (now termed MASH) was associated with increased overall mortality compared with the general population and that cardiovascular disease was a leading cause of death [30]. However, prior evidence regarding the prevalence and correlation of high-risk MASH with cardiovascular comorbidities has largely relied on small, biopsy-based cohorts, which may suffer from selection bias and limited generalizability. Our study utilized the FAST score to characterize high-risk MASH within the large nationally representative NHANES dataset, effectively moving the observation from the clinic to the community. To our knowledge, this is the first large-scale study to demonstrate a significant association between the non-invasive identification of high-risk MASH and prevalent IHD. Our findings extend the existing literature by moving beyond the broad spectrum of MASLD to a specific subgroup with markers of active fibrotic steatohepatitis [15, 27].

In our analysis of NHANES participants, using different cutoff values of FAST score for high-risk MASH, our study demonstrated that the prevalence of IHD among individuals with high-risk MASH (10.5%–11.5%) was significantly lower than that reported in hospital-based NAFLD/MASLD cohorts (55.4%; 95% CI: 39.6%–70.1%) [12]. The difference in results is likely due to the different selection of the population (a real-world population instead of hospital-based cohorts) and the different study designs (cross-sectional rather than a longitudinal study). However, notably, the strength of the association we observed for high-risk MASH (OR: 2.08; 95% CI: 1.18–3.65) exceeded that typically reported for the broader spectrum of NAFLD/MASLD (OR: 1.33; 95% CI: 1.21–1.45) [12]. This finding supports the hypothesis that the high-risk MASH carries a substantially greater cardiovascular burden than simple steatosis, warranting distinct risk stratification.

While liver fibrosis is a well-established predictor of liver-related mortality [15, 27], our data suggest that a significant association with prevalent IHD is pronounced in high-risk MASH, a condition characterized by the coexistence of marked inflammation and fibrosis, as jointly reflected by the FAST score. This perspective is consistent with evidence indicating that cardiovascular risk in MASLD/MASH may reflect not only fibrosis burden but also inflammatory activity and cardiometabolic dysfunction [14, 27]. 

Our component analysis of the FAST score provides further mechanistic insight into the observed relationship between high-risk MASH and IHD. We found that both LSM and CAP retained significant independent associations with IHD, whereas AST did not. This finding aligns with previous observations suggesting that structural hepatic changes and metabolic fat burden may exhibit stronger correlations with cardiovascular prevalence than enzymatic markers of inflammation alone [29, 31]. The independent association of LSM with IHD in our study is consistent with accumulating data linking liver fibrosis to coronary atherosclerosis [32]. Specifically, our results mirror those of Park et al., who demonstrated in a cohort of NAFLD patients who liver stiffness measured by magnetic resonance elastography (MRE) was independently associated with high-risk cardiovascular disease [33]. These associations likely reflect the shared pathophysiological milieus between MASH and IHD. As reviewed by Muzurović et al., NAFLD and cardiovascular disease share a cluster of cardiometabolic risk factors, including insulin resistance, oxidative stress, and chronic low-grade inflammation [34]. In this context, elevated liver stiffness may act as an integrator of cumulative inflammatory damage and endothelial dysfunction, which are central to the progression of both hepatic and coronary atherosclerosis. Similarly, the independent relationship between CAP and IHD highlights the role of hepatic lipid burden in metabolic dysregulation. Xi and Yang recently reported in the NHANES 2017–2020 cohort that elevated CAP values were significantly associated with a higher cardiometabolic index (CMI), a novel indicator of lipid dysfunction and vascular risk [35]. Consistent with their findings, our data suggest that the lipotoxic burden captured by CAP adds a distinct layer of cardiovascular risk information that is not fully encompassed by fibrosis markers alone.

Collectively, these findings validate the rationale behind using the FAST score—a composite algorithm—to define high-risk MASH. Since the pathogenesis of IHD in MASLD involves both profibrotic pathways (mediating vascular remodeling) and lipotoxic pathways (driving systemic insulin resistance), a composite score such as FAST that integrates both dimensions offers a more holistic risk assessment than single biomarkers alone. By combining markers of structural remodeling (LSM) and metabolic burden (CAP), the FAST score identifies a phenotype characterized by a “double burden” of liver pathology. In this cross-sectional setting, identifying individuals with elevated FAST scores serves not only to flag those at risk for progressive MASLD but also to highlight a subgroup with a significantly higher prevalence of IHD.

Our findings revealed a significant association between high-risk MASH and HF. HF represents the end stage of various cardiovascular diseases, particularly IHD. Moreover, a well-established link exists between the broad spectrum of MASLD/NAFLD and an increased risk of HF [36, 37], making this result somewhat anticipated. Moreover, we observed no significant association between high-risk MASH and stroke. A study based on 337 patients also demonstrated no significant difference in stroke risk between patients with MASH and those with simple steatosis [38]. Further investigations are warranted to clarify the potential relationship between MASH and stroke.

Collectively, these findings underscore the necessity for a multidisciplinary approach to patient management. Identification of high-risk MASH via the FAST score should alert clinicians not only to the potential for hepatic progression but also to a significantly elevated burden of concurrent IHD. Additionally, patients identified as high-risk MASH by this non-invasive tool warrant vigilant cardiovascular risk assessment and aggressive optimization of metabolic risk factors. Future prospective studies are needed to confirm whether the FAST score can independently predict incident cardiovascular events in the general population and whether therapeutic resolution of high-risk MASH translates into a tangible reduction in cardiovascular morbidity.

### Potential sex-specific differences in the high-risk MASH–IHD association

Our exploratory analysis suggests that sex may affect the association between high-risk MASH and IHD (P for interaction = 0.035). A strong positive association was observed in males, whereas no such relationship was evident in females. This divergence aligns with data from a large retrospective analysis of the U.S. National Readmission Database, including 41,005 MASH/NASH patients, which demonstrated that females had 54% lower odds of coronary artery disease than their male counterparts after adjustment for age (OR: 0.46, 95% CI: 0.44–0.49) [39]. However, we urge caution in interpreting these findings, as several factors suggest they should be viewed as hypothesis-generating rather than conclusive.

First, from a statistical perspective, the observed interaction (*p* = 0.035) is of borderline significance, and the confidence interval of effect size is relatively wide in females (OR: 0.36; 95% CI: 0.07–1.27). This instability likely reflects the limited number of IHD cases in the high-risk female group. Increased statistical power would enable more precise effect size estimation within the female subgroup and could either confirm or refute the current findings. Future studies with large sample sizes and/or prospective designs could help clarify whether this interaction represents a true biological difference or is a consequence of statistical variability. Second, potential diagnostic bias may have contributed to the null finding in women. Evidence suggests that women often present with atypical IHD symptoms and are less likely to undergo standard diagnostic procedures, leading to underdiagnosis [40]. The Women’s Ischemia Syndrome Evaluation (WISE) study highlighted that women frequently suffer from coronary microvascular dysfunction, a condition often missed by conventional angiography used to define IHD in many clinical settings [41]. This potential diagnostic bias could have attenuated a true positive association between high-risk MASH and IHD in women, thereby contributing to the observed sex difference. Therefore, we cannot rule out the possibility that the association exists in women but was masked by both limited statistical power and potential diagnostic bias. The observed sex difference should be considered preliminary and emphasize the need for future studies with larger cohorts specifically designed to investigate sex differences in MASH and IHD.

Biologically, such disparities might reflect the cardioprotective properties of estrogen or sex-specific patterns of visceral adiposity, which are known to influence systemic inflammation and insulin resistance [42–44]. However, given the limitations of statistical power and potential diagnostic underascertainment of IHD in women, these findings must be interpreted as hypothesis-generating. Definitive conclusions regarding sex-specific risks will require validation in large-scale prospective cohorts specifically designed to capture sufficient cardiovascular events in female populations.

### Clinical implications and mechanistic insights

Our study extends the current literature by offering a pragmatic, population level evaluation of high-risk MASH using the FAST score—a validated non-invasive tool integrating LSM, CAP, and AST [15]. To our knowledge, this is the first study to investigate the association between the FAST score and cardiovascular outcomes in a nationally representative cohort. Unlike previous research that relied on invasive liver biopsies or simple fibrosis indices (e.g., FIB-4 or NFS), our approach leverages a composite biomarker specifically designed to identify patients with active fibrotic MASH [13]. This metric offers a distinct advantage: it captures not only fibrosis but also ongoing hepatic necroinflammatory activity, providing a more holistic assessment of progressive liver disease risk. By utilizing this refined definition, we were able to identify a specific high-risk MASH within the general population that carries a disproportionately elevated burden of IHD, a distinction that broader MASLD definitions often obscure. This approach aligns with emerging strategies in precision medicine that utilize population-based scoring systems to better stratify individual vascular health risks [45].

The association observed between high-risk MASH and IHD is likely underpinned by multifactorial mechanisms. First, the association between high-risk MASH and IHD may be explained by shared cardiometabolic risk factors. A multinational cohort study of biopsy-confirmed NAFLD patients observed that individuals with advanced fibrosis (F3-F4) faced a significant burden of vascular events, and diabetes, larger BMI, older age, and higher low-density lipoprotein cholesterol levels were positively associated with the occurrence of IHD [27]. This aligns with the common soil hypothesis, suggesting that hepatic and cardiac pathologies share a systemic metabolic foundation. Indeed, recent NHANES-based analyses reinforced this metabolic link by demonstrating that a lower estimated glucose disposal rate (eGDR)—a proxy for insulin resistance—is strongly associated with the severity of liver fibrosis and MASLD [46]. Consistent with this, our study found that participants identified as high-risk MASH had a significantly higher prevalence of dyslipidemia, hypertension, diabetes, and obesity compared to those without high-risk MASH. Second, beyond shared metabolic comorbidities, the specific pathological components of high-risk MASH—namely hepatic fibrosis and inflammation—appear to be intrinsically linked to cardiovascular health. Hepatic fibrosis has been identified as a predictor of long-term CVD risk in patients with MASLD [15, 47]. Recent evidence indicates that advanced biopsy fibrosis and a higher fibrosis score are significantly correlated with the presence of cardiovascular disease [48]. Furthermore, a heightened systemic inflammatory response, which characterizes progressive MASH, has been reported to be closely linked to the pathophysiology of IHD [49]. Recent studies have increasingly emphasized that inflammatory and profibrotic activity in MASH, rather than hepatic steatosis alone, may contribute to atherosclerotic risk [50–52]. This evolving paradigm underscores the complex interplay between metabolic dysfunction, chronic inflammation, and vascular pathology. Our findings highlight a potential link between hepatic and cardiovascular health and suggest that the FAST score, beyond its primary role in hepatology, may also help identify individuals characterized by a dual burden of hepatic and metabolic risk. While these associations are intriguing, we must interpret them with caution given the cross-sectional nature of our study. It remains to be determined whether the FAST score offers incremental value over established cardiovascular risk calculators.

Nevertheless, in the emerging era of MASH pharmacotherapy, marked by the recent FDA approval of Resmetirom [53] and promising data for Semaglutide [54], identifying high-risk MASH patients has become increasingly important. Concurrently, as highlighted by Mubeen et al., the expanding role of SGLT2 inhibitors in preventing heart failure and adverse vascular events offers a complementary therapeutic strategy, reinforcing the need for a holistic cardiometabolic approach in these patients [55]. Our data raise the possibility that patients identified as high-risk by the FAST score might benefit from comprehensive cardiovascular risk assessment, although this strategy requires rigorous validation in prospective cohorts. A critical unanswered question is whether resolving MASH through these novel therapies could concomitantly reduce the incidence of IHD and other cardiovascular events. Thus, our study serves as a hypothesis-generating step, providing a rationale for longitudinal investigations into the potential synergy between MASH resolution and cardiovascular risk reduction.

### Strengths and limitations

The current study has several strengths. First, to our knowledge, this is the first study to specifically investigate the association between high-risk MASH—as defined by the FAST score—and the odds of IHD. While previous literature has broadly explored the cardiovascular risks of generic MASLD, the specific relationship between this active fibrotic phenotype (identified via the FAST score) and IHD has not been previously reported. By utilizing the FAST score to screen patients with significant fibrosis and inflammatory activity from those with simple steatosis, our study moves beyond broad disease definitions. This targeted approach highlights the distinct and elevated association between active fibrotic MASH features—rather than steatosis alone—and the prevalence of ischemic heart disease. Second, the utilization of the FAST score represents a significant methodological refinement. Unlike prior research relying on invasive biopsy or simple imaging (which fail to distinguish MASH from simple steatosis), the FAST score is a validated composite biomarker integrating liver fat (CAP), stiffness (LSM) and inflammation (AST). This allows for a more granular risk stratification at the population level, capturing a patient subgroup with greater clinical severity and metabolic burden. Finally, the results of this study were based on the NHANES 2017–2018 dataset, a large, national survey whose results generalize well to the nonhospitalized U.S. adult population.

However, several limitations must be acknowledged. First, the cross-sectional design of the NHANES inherently precludes the establishment of temporal relationships or causal inference. While we identified an association between the prevalence of high-risk MASH and IHD, we cannot determine the disease onset sequence. Furthermore, although we adjusted for a comprehensive array of potential confounders, residual confounding remains a possibility. Specifically, NHANES lacks granular data on IHD severity (e.g., prior revascularization procedures), specific stroke subtypes, or the duration of medication use, all of which could influence the estimated strength of the association. Second, although the overall sample size was large, the number of participants identified with high-risk MASH was relatively small (*n* = 264), particularly when stratified by sex (*n* = 31). This limitation resulted in wider confidence intervals for sex-specific estimates, necessitating that our findings regarding sex differences be interpreted as preliminary and hypothesis-generating. Third, potential selection bias or survivor bias cannot be ruled out. Given the cross-sectional nature of the study, individuals with the most severe forms of MASH or rapidly fatal cardiovascular events may have had higher mortality rates or been institutionalized, preventing their inclusion in the NHANES survey. This exclusion of the most severe cases could theoretically lead to underestimation of the true strength of the association in the general population. Finally, while the FAST score is a well-validated screening tool, it is not a diagnostic gold standard. The gold standard remains liver biopsy. Its derivation in specialist hepatology cohorts with high disease prevalence means that its performance characteristics in a general population setting may vary. To mitigate this, we employed dual cutoffs (high-sensitivity and high-specificity), and the persistence of the IHD association across both thresholds strengthens our confidence that the findings are not artifacts of misclassification. Nevertheless, future validation using biopsy-proven cohorts or emerging imaging biomarkers remains desirable.

## Conclusion

In summary, this study provides nationally representative evidence demonstrating an independent association between high-risk MASH—identified by the non-invasive FAST score—and IHD in the U.S. population. Our findings move beyond the broad categorization of MASLD to specifically implicate the high-risk phenotype, characterized by active fibrotic steatohepatitis, as a significant correlate of cardiovascular comorbidity. Future prospective longitudinal studies are essential to validate these cross-sectional observations and determine whether resolving high-risk MASH translates into improved cardiovascular outcomes.

## Supplementary Information

Supplementary Material 1.

## Data Availability

The datasets generated and/or analyzed in this study are available from the NHANES repository (https://www.cdc.gov/nchs/nhanes/).

## Abbreviations

IHD: Ischemic heart disease
HF: Heart failure
MASLD: Metabolic dysfunction-associated steatotic liver disease
MASH: Metabolic dysfunction-associated steatohepatitis
NAFLD: Nonalcoholic fatty liver disease
NASH: Nonalcoholic steatohepatitis
NAS: NAFLD activity score
CVD: Cardiovascular disease
FAST: Fibroscan-AST
CAP: Controlled attenuation parameter
LSM: Liver stiffness measurement
AST: Aspartate aminotransferase
F: Fibrosis
CSF: Clinically significant fibrosis
VCTE: Vibration controlled transient elastography
NHANES: National Health and Nutrition Examination Survey
CDC: Centers for Disease Control and Prevention
SBP: Systolic blood pressure
DBP: Diastolic blood pressure
BMI: Body mass index
CRP: C-reactive protein
ALT: Alanine aminotransferase
eGFR: Estimated glomerular filtration rate
HbA1c: Glycated hemoglobin
TG: Triglyceride
BUN: Blood urea nitrogen
TC: Total cholesterol
HDL-C: High-density lipoprotein cholesterol
OR: Odds ratio
CI: Confidence interval
MCMC: Markov chain Monte Carlo
WISE: Women’s Ischemia Syndrome Evaluation
CMI: Cardiometabolic index
MRE: Magnetic resonance elastography
eGDR: Estimated glucose disposal rate
