# Supplementary material for: Association between high-risk metabolic dysfunction-associated steatohepatitis and ischemic heart disease: a nationally representative study
Source: Nutr J. 2026 Jun 2;25:85. doi: 10.1186/s12937-026-01342-6 (PMC13455423; doi:10.1186/s12937-026-01342-6)
Supplement: Supplementary file 1 [file 12937_2026_1342_MOESM1_ESM.docx]

**Data Supplement**

***Association between high-risk metabolic dysfunction-associated steatohepatitis and ischemic heart disease: A nationally representative study***

**Table of Contents**

| Table S1 | Clinical characteristics of included and excluded subjects | P3 |
| --- | --- | --- |
| Table S2 | Sensitive analysis of the association between high-risk MASH (FAST score ≥ 0.35) with risk of IHD in adult Americans based on the multiple-imputation analysis | P5 |
| Table S3 | Association of high-risk MASH (FAST score ≥ 0.25) with ischemic heart disease in adult Americans from the National Health and Nutrition Examination Survey 2017-2018 | P6 |
| Table S4 | Association of high-risk MASH (FAST score ≥ 0.35) with risk of diseases in adult Americans from the National Health and Nutrition Examination Survey 2017-2018 | P7 |
|  |  |  |

**Table S1: Clinical characteristics of included and excluded subjects**

| **Variables** | **Total subjects**  **N=5856** | **Excluded subjects**  **N=2160** | **Included subjects**  **N=3696** | ***p*** |
| --- | --- | --- | --- | --- |
| Demographic variables |  |  |  |  |
| Age, year | 47.26(46.20,48.38) | 45.09(43.65,46.54) | 48.27(46.88,49.66) | 0.006 |
| Male, sex | 48.21(46.39,50.03) | 50.00(45.53,54.47) | 47.41(45.02,49.81) | 0.346 |
| Race/ethnicity |  |  |  | 0.161 |
| Mexican American | 10.84(7.36,15.68) | 12.17(8.22,17.65) | 9.68(6.55,14.08) |  |
| Other Hispanic | 7.27(5.83,9.03) | 7.33(5.96,8.98) | 7.23(5.56,9.33) |  |
| Non-Hispanic White | 59.13(53.47,64.56) | 55.29(48.93,61.49) | 62.49(57.03,67.65) |  |
| Non-Hispanic Black | 11.85(8.79,15.79) | 13.35(9.96,17.68) | 10.53(7.55,14.50) |  |
| Other race | 10.91(8.61,13.74) | 11.85(9.50,14.69) | 10.08(7.57,13.32) |  |
| Comorbidities |  |  |  |  |
| Physical activity |  |  |  | 0.968 |
| Below guidelines | 34.13(32.40,35.90) | 35.46(32.36,38.67) | 33.54(31.33,35.82) |  |
| Meets guidelines | 13.95(12.58,15.45) | 12.68(10.42,15.34) | 14.51(12.98,16.20) |  |
| Exceeds guidelines | 51.92(50.11,53.73) | 51.86(48.99,54.72) | 51.95(49.33,54.56) |  |
| Smoking status |  |  |  | 0.052 |
| Current | 17.07(14.72,19.70) | 20.34(16.25,25.14) | 15.62(13.01,18.63) |  |
| Former | 24.06(22.18,26.05) | 23.20(21.15,23.38) | 24.44(21.69,27.42) |  |
| Never | 58.87(55.44,62.23) | 56.46(51.22,61.57) | 59.94(56.38,63.39) |  |
| Hypertension | 37.26(34.00,40.64) | 36.99(33.53,40.59) | 38.46(34.36,42.73) | 0.867 |
| Systolic BP, mm Hg | 122(121,123) | 122(121,124) | 121(120,123) | 0.996 |
| Diastolic BP, mm Hg | 72(71,73) | 71(69,73) | 73(72,75) | 0.099 |
| BMI, kg/m2 | 29.70(29.16,30.24) | 28.95(28.42,29.48) | 30.03(29.46,30.60) | <0.001 |
| Obesity | 41.95(38.22,45.77) | 36.79(33.01,40.74) | 44.18(40.06,48.39) | <0.001 |
| Diabetes | 12.92(11.79,14.14) | 10.89(9.69,12.22) | 15.50(14.03,17.09) | <0.001 |
| Laboratory variables |  |  |  |  |
| HbA1c(%) | 5.67(5.64,5.71) | 5.55(5.51,5.59) | 5.72(5.68,5.76) | <0.001 |
| Total cholesterol, mg/dL | 188.12(184.68,191.56) | 187.34(182.94,191.74) | 188.41(184.78,192.04) | 0.599 |
| Triglyceride | 111.13(104.90,117.36) | 108.53(96.57,120.50) | 112.11(105.27,118.95) | 0.600 |
| HDL-C, mg/dL | 53.56(52.60,54.51) | 56.37(54.71,58.03) | 52.52(51.72,53.33) | <0.001 |
| Serum creatinine, mg/dL | 0.88(0.87,0.89) | 0.87(0.85,0.89) | 0.88(0.87,0.90) | 0.143 |
| eGFR | 94.76(93.17,96.36) | 97.66(95.73,99.58) | 95.96(94.21,97.70) | 0.004 |
| ALT, IU/L | 22.89(22.14,23.65) | 24.20(22.44,25.95) | 22.42(21.69,23.15) | 0.076 |
| Serum total albumin (g/L) | 4.08(4.05,4.12) | 4.07(4.03,4.10) | 4.09(4.06,4.12) | 0.132 |
| CRP | 3.90(3.60,4.21) | 3.79(3.36,4.21) | 3.95(3.59,4.31) | 0.540 |
| AST, IU/L | 22.19(21.66,22.72) | 24.54(23.11,25.96) | 21.34(20.92,21.77) | <0.001 |

*Abbreviations*: BMI, body mass index; BP, blood pressure; HbA1c, glycated hemoglobin; HDL-C, high density lipoprotein cholesterol; eGFR, estimated glomerular filtration rate; AST, aspartate aminotransferase; ALT, alanine Aminotransferase; CRP, C-reactive protein.

**Table S2: Sensitive analysis of the association between high-risk MASH (FAST score ≥ 0.35) with risk of IHD in adult Americans based on the multiple-imputation analysis**

| Dataset | Odds ratio | 95% confidence interval | P value |
| --- | --- | --- | --- |
| Data 1 | 1.87 | 1.08,3.19 | 0.022 |
| Data 2 | 1.86 | 1.07,3.18 | 0.023 |
| Data 3 | 1.87 | 1.08,3.19 | 0.023 |
| Data 4 | 1.86 | 1.07,3.16 | 0.024 |
| Data 5 | 1.86 | 1.08,3.17 | 0.024 |
| Pool | 1.86 | 1.08,3.20 | 0.024 |

Analysis was adjusted sex, race, age, physical activity, smoking status, BMI, diabetes mellitus, hypertension, antihypertensive drugs, glucose-lowering drugs, cholesterol-lowering drugs, eGFR, TC, HDL-C, ALT, serum total protein, CRP.

*Abbreviations*: MASH, metabolic dysfunction-associated steatohepatitis; FAST, FibroScan-AST; BMI, body mass index; eGFR, estimated glomerular filtration rate; TC, total cholesterol; HDL-C, high density lipoprotein cholesterol; ALT, alanine aminotransferase; CRP, C-reactive protein.

**Table S3: Association of high-risk MASH (FAST score ≥ 0.25) with ischemic heart disease in adult Americans from the National Health and Nutrition Examination Survey 2017-2018**

|  | **Cases/N** | **Odds ratio** | **95% confidence interval** | **P value** |
| --- | --- | --- | --- | --- |
| FAST score < 0.25 | 229/3242 | Ref | Ref | Ref |
| FAST score ≥ 0.25 | 46/454 |  |  |  |
| Crude |  | 1.48 | 1.06,2.07 | 0.020 |
| Model1 |  | 1.45 | 1.01,2.07 | 0.044 |
| Model2 |  | 1.77 | 1.15,2.73 | 0.010 |
| Model3 |  | 1.65 | 1.03,2.64 | 0.038 |

*Note*: Model 1 was adjusted sex, race, and age; Model 2 was adjusted sex, race, age, physical activity and smoking status; Model 3 was adjusted sex, race, age, physical activity, smoking status, BMI, diabetes mellitus, hypertension, antihypertensive drugs, glucose-lowering drugs, cholesterol -lowering drugs, eGFR, TC, HDL-C, ALT, serum total protein, CRP.

*Abbreviations*: MASH, metabolic dysfunction-associated steatohepatitis; FAST, FibroScan-AST; BMI, body mass index; eGFR, estimated glomerular filtration rate; TC, total cholesterol; HDL-C, high density lipoprotein cholesterol; ALT, alanine aminotransferase; CRP, C-reactive protein.

**Table S4: Association of high-risk MASH (FAST score ≥ 0.35) with risk of diseases in adult Americans from the National Health and Nutrition Examination Survey 2017-2018**

| **FAST score** | **Cases/N** | **OR (95% CI), P value** | |  |  |
| --- | --- | --- | --- | --- | --- |
|  |  | **Crude model** | **Model 1** | **Model 2** | **Model 3** |
| HF |  |  |  |  |  |
| FAST score < 0.35 | 80/3432 | Ref | Ref | Ref | Ref |
| FAST score ≥ 0.35 | 17/264 | 3.43(1.96,5.99), <0.001 | 3.60(2.02,6.43), <0.001 | 3.57(1.95,6.23), <0.001 | 3.05(1.44,6.24), 0.003 |
| Stroke |  |  |  |  |  |
| FAST score < 0.35 | 149/3432 | Ref | Ref | Ref | Ref |
| FAST score ≥ 0.35 | 9/264 | 0.84(0.42,1.67), 0.615 | 0.99(0.46,2.00), 0.98 | 0.98(0.45,1.88), 0.96 | 0.80(0.33,1.73), 0.57 |

*Note*: Model 1 was adjusted sex, race, and age; Model 2 was adjusted sex, race, age, physical activity and smoking status; Model 3 was adjusted sex, race, age, physical activity, smoking status, BMI, diabetes mellitus, hypertension, antihypertensive drugs, glucose-lowering drugs, cholesterol -lowering drugs, eGFR, TC, HDL-C, ALT, serum total protein, CRP.

*Abbreviations*: MASH, metabolic dysfunction-associated steatohepatitis; FAST, FibroScan-AST; BMI, body mass index; eGFR, estimated glomerular filtration rate; TC, total cholesterol; HDL-C, high density lipoprotein cholesterol; ALT, alanine aminotransferase; CRP, C-reactive protein.
